# COVID-19 classification using chest X-ray images: A framework of CNN-LSTM and improved max value moth flame optimization

**DOI:** 10.3389/fpubh.2022.948205

**Published:** 2022-08-30

**Authors:** Ameer Hamza, Muhammad Attique Khan, Shui-Hua Wang, Abdullah Alqahtani, Shtwai Alsubai, Adel Binbusayyis, Hany S. Hussein, Thomas Markus Martinetz, Hammam Alshazly

**Affiliations:** ^1^Department of Computer Science, HITEC University, Taxila, Pakistan; ^2^Department of Mathematics, University of Leicester, Leicester, United Kingdom; ^3^College of Computer Engineering and Sciences, Prince Sattam Bin Abdulaziz University, Al-Kharj, Saudi Arabia; ^4^Department of Electrical Engineering, College of Engineering, King Khalid University, Abha, Saudi Arabia; ^5^Department of Electrical Engineering, Faculty of Engineering, Aswan University, Aswan, Egypt; ^6^Institute for Neuro- and Bioinformatics, University of Lübeck, Lübeck, Germany; ^7^Faculty of Computers and Information, South Valley University, Qena, Egypt

**Keywords:** coronavirus, enhancement, deep learning, LSTM, optimization

## Abstract

Coronavirus disease 2019 (COVID-19) is a highly contagious disease that has claimed the lives of millions of people worldwide in the last 2 years. Because of the disease's rapid spread, it is critical to diagnose it at an early stage in order to reduce the rate of spread. The images of the lungs are used to diagnose this infection. In the last 2 years, many studies have been introduced to help with the diagnosis of COVID-19 from chest X-Ray images. Because all researchers are looking for a quick method to diagnose this virus, deep learning-based computer controlled techniques are more suitable as a second opinion for radiologists. In this article, we look at the issue of multisource fusion and redundant features. We proposed a CNN-LSTM and improved max value features optimization framework for COVID-19 classification to address these issues. The original images are acquired and the contrast is increased using a combination of filtering algorithms in the proposed architecture. The dataset is then augmented to increase its size, which is then used to train two deep learning networks called Modified EfficientNet B0 and CNN-LSTM. Both networks are built from scratch and extract information from the deep layers. Following the extraction of features, the serial based maximum value fusion technique is proposed to combine the best information of both deep models. However, a few redundant information is also noted; therefore, an improved max value based moth flame optimization algorithm is proposed. Through this algorithm, the best features are selected and finally classified through machine learning classifiers. The experimental process was conducted on three publically available datasets and achieved improved accuracy than the existing techniques. Moreover, the classifiers based comparison is also conducted and the cubic support vector machine gives better accuracy.

## Introduction

In December 2019, Wuhan, Hubei Province, China, became the epicenter of an unknown-cause pneumonia epidemic, attracting national and international attention (1). The current outbreak of coronavirus disease 2019 (COVID-19), a coronavirus-associated acute respiratory illness, is the third worldwide pandemic in less than two decades (2). The COVID-19 sickness is caused by the SARS-CoV-2 virus (3). In severe cases, this illness can cause organ failure and breathing difficulties. Aside from the medical consequences, the disease had a massive economic and environmental impact on the world (4, 5).

Coronavirus disease 2019 detection methods include nucleic acid-based assays and polymerase chain reaction (PCR). The traditional real-time polymerase chain reaction (RT-PCR) method of COVID-19 detection is time-consuming (6). Artificial intelligence (AI) technologies have been widely used to combat the COVID-19 outbreak and its complications. To identify the COVID-19 instance based on the X-ray images, an automation method is required. It is the least expensive process when compared to the COVID-19 test. Human examination of these photographs, on the other hand, is a difficult and time-consuming task. For accurate classification, an expert physician is always required. As a result, it is critical to find these photos as soon as possible using a reliable method. In clinics, computerized approaches assist radiologists in confirming their subjective results and detecting COVID-19 (7).

The AI-based estimation methods rely on data from the patient's symptoms. A person infected with the coronavirus usually exhibits no signs or symptoms. As a result, identifying an infectious individual is extremely difficult (8). Traditional feature-based approaches and deep learning-based techniques are the two categories of AI-based techniques. Traditional features-based algorithms include some preprocessing procedures, handcrafted features (such as shape, texture, and geometric characteristics), removal of extraneous features, and classification. In deep learning architectures, raw photos are fed into convolutional neural network (CNN) models, which extract features from convolution layers and perform classification using the fully connected layers. Following that, a few researchers used feature optimization methods to select the best features before classifying them with the Softmax classifier.

Using deep learning (DL), several techniques are introduced for COVID-19 diagnosis and classification using chest X-rays and CT images (9–15). Additionally, CNN models are useful in the deployment of sophisticated COVID-19 pneumonia detection systems (16). Numerous strategies for identifying COVID-19 have been presented, all of which make use of deep CNN features and generate more precise findings than manual feature-based methods (17). In a few studies, the researchers focused on feature fusion techniques to get better information about an image. They fused features from different sources into one feature matrix. Özkaay et al. (18) fused deep features for COVID-19 classification using the feature ranking method. Shankar et al. (19) introduced an entropy based handcrafted and deep features fusion approach for better classification of COVID-19. Ragab et al. (20) combined several features based on concatenated fashion. These techniques performed well in terms of accuracy but on the other side, the computational time is significantly increased. Few researchers introduced feature reduction techniques to resolve the problem of high computational time but the reduction process decreases in accuracy due to dropping some important features (21, 22).

Feature selection is an important research area nowadays and many techniques are introduced in the literature. As compared to features reduction techniques, the feature selection technique is the process of subset selection from the originally extracted features instead of generating new features. The purpose of feature selection techniques is to reduce the computational time by selecting only important features based on some selection criteria or fitness function. A few important feature selection techniques are- genetic algorithm based selection, particle swarm optimization based selection, entropy based selection, bee colony optimization based selection, and many more (23).

In recent years, many research works have been done for the detection and classification of COVID-19 in X-ray and CT-scan images (24). They followed some traditional techniques and showed improved accuracy (25); however, COVID-19 patients are increasing day by day worldwide. A lot of data has been generated in the form of Chest X-ray and CT images that are not feasible for classification through traditional techniques. The traditional techniques work better for the smaller datasets but for the large datasets, accuracy is degraded (26). Based on this reason, it is room for improving the accuracy through the development of deep learning architectures. In this article, we proposed a new architecture based on deep learning and improved moth flame optimization for COVID-19 classification. Our major contributions are as follows:

- A contrast enhancement technique is proposed based on the fusion of the output of local and global filters. The resultant enhanced image is further utilized for the augmentation process.
- Proposed a CNN-LSTM architecture and trained it using deep transfer learning from scratch instead of freezing a few layers.
- Proposed a new features fusion technique named Serial based Maximum Information.
- An improved max value based moth flame optimization algorithm is proposed for best features selection.

## Related study

Many computerized techniques have been introduced for COVID-19 in recent years by researchers of computer vision (27). Several researchers focused on traditional techniques and few of them using deep learning architectures for the detection and classification of COVID-19 from chest X-ray images. Ibrahim et al. (28) presented a deep learning method for multiclass classification problems such as COVID-19, pneumonia, and normal. They used a pre-trained CNN model named AlexNet and trained it on selected COVID-19 datasets. They considered the problems of both binary and multiclass and achieved accuracies of 94.43, 98.19, and 95.78%, respectively. The limitation of this study was the lack of data for training. Ismael and Sengür (24) presented a deep-learning-based technique for classifying COVID-19 and normal (healthy) chest X-ray images. They followed some sequential steps including deep feature extraction, fine-tuning of pre-trained CNNs, and end-to-end training of a fine-tuned CNN model. Three pre-trained CNN models were used for the training and feature extraction such as ResNet18, VGG16, and VGG19. The extracted deep features were finally classified using the Support Vector Machines (SVM) classifier. The fine-tuned ResNet50 deep model gives better accuracy of 92.60% than the other methods. The drawback of this method was less number of training samples. Ketu and Mishra (29) introduced a CNN-LSTM deep learning model that can accurately detect the COVID-19 infection. The proposed approach extracts useful information from the convolutional layers. Later on, the Long short-term memory (LSTM) network is designed to extract features that are fused with CNN features. The limitation of the presented method was the reliability and suitability of the model to the other series of data. Nivetha et al. (30) presented a new classification technique for COVID-19 based on Neighborhood Rough Neural Network Algorithm (NRNN). The presented method performed better than existing algorithms like Backpropagation Neural Network (BNN), Decision Tree, and Naive Bayes Classifiers. The accuracies of NRNN were 98, 92, 100, and 100% which was significantly better than other methods. Moreover, NRNN consumes less amount of training data compared to the existing methods. Shastri et al. (31) introduced a novel neural network based framework for COVID-19 classification. They used ChestXImageNet CNN model for the classification purpose and tested on the open-access dataset that consisted of both binary classes and multiclass and achieved accuracies of 100 and 100%, respectively. Khan et al. (32) described a deep learning technique in which they used three pre-trained models named EfficientNet B1, NasNetMobile, and MobileNetV2. Before training deep models, they performed data augmentation. Moreover, they optimized hyper-parameters for improving accuracy. The described model achieved 96.13% accuracy which was better than the existing methods. The limitation of described work was the use of high-weighted models that required high time for computation.

Imagawa et al. (33) presented a hybrid framework for the classification of COVID-19 images. They used two pre-trained deep learning models named- AlexNet and ResNet34 with and without transfer learning. On both fine-tuned models, classification is performed and attained improved accuracy. Falco et al. (34) designed another evolutionary algorithm based approach for COVID-19 classification. Sarki et al. (35) developed a deep learning system for the classification and valid detection of coronavirus using chest images. They evaluated the traditional networks and also developed a CNN from scratch and trained on the binary class and multiclass based datasets. Öztürk et al. (36) designed a machine learning method for the classification of viral epidemics by analyzing chest X-ray images and CT images. They applied hand-crafted feature extraction to make the data more convenient and optimized the features by using stacked auto-encoder and principal component analysis techniques. Al-Zubaidi et al. (37) applied CNNs for the classification of COVID-19 images. They used Google-Net for training and extracting automated features from the images. The above methods have several gaps such as—not performing well on imbalanced datasets and increasing higher computational time. Shazia et al. (38) presented a neural network based system for COVID-19 detection from Chest X-Ray images. They used three pre-trained models and fine-tuned them. The fine-tuned models have been trained through transfer learning and obtained improved accuracy. Shazia et al. (39) presented a comparative study of several deep learning models for COVID-19 classification from Chest X-Ray images. They used seven pre-trained deep models such as VGG16 and ResNet50 and named a few more and attained a classification accuracy of 99.48%. Joloudari et al. (40) combined the CNN model with a global feature extractor for the classification of COVID-19 infected and healthy patients. They used 10-fold cross-validation and obtained an accuracy of 96.71%.

## Proposed methodology

**Figure 1:**
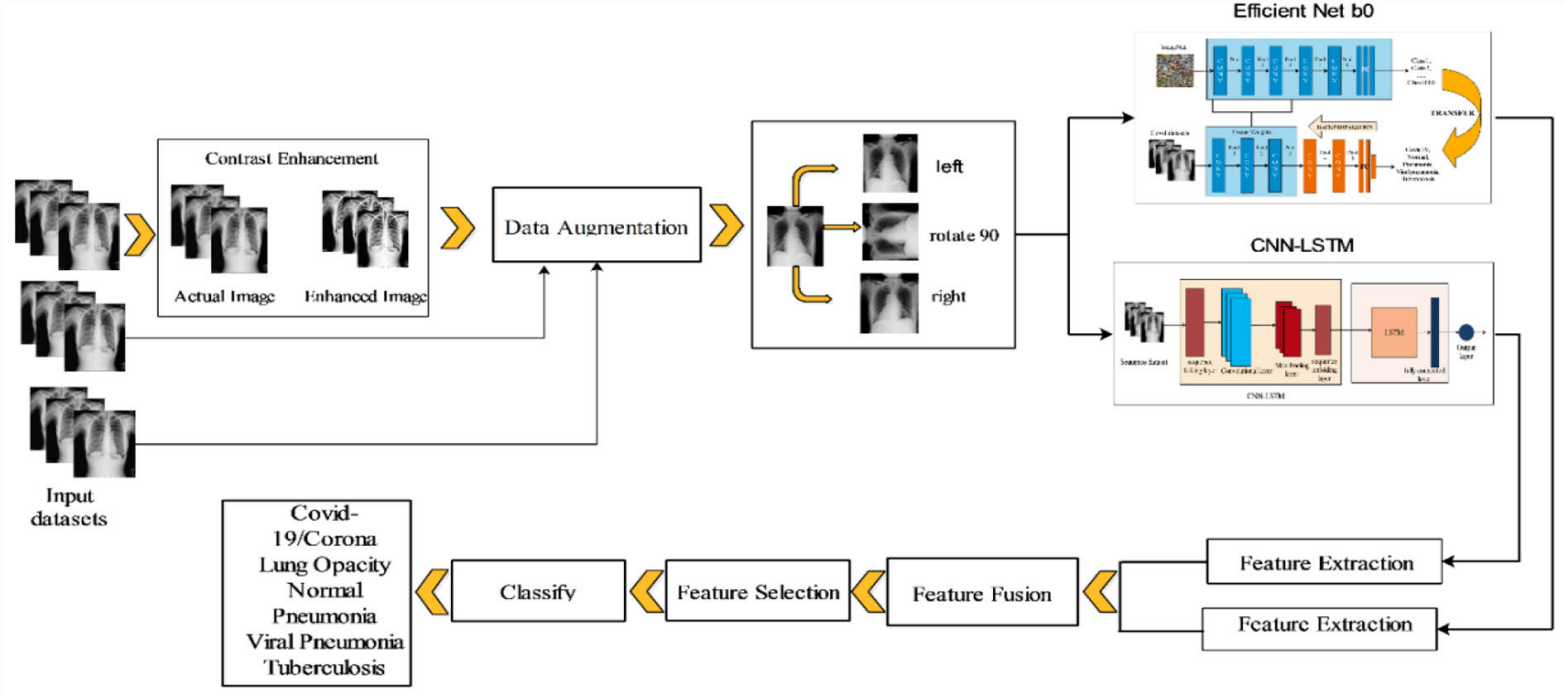
The proposed architecture of coronavirus disease 2019 (COVID-19) classification using an efficient network and CNN-LSTM.

Figure 1 depicts the suggested CNN-LSTM deep learning and features optimization architecture. In this diagram, the original images are acquired and the contrast is enhanced using a combination of filtering algorithms. Then, to expand the size of the dataset, data augmentation is used to train two deep learning networks: Modified EfficientNet B0 and CNN-LSTM. Both networks are built from scratch and extract information from the deep layers. Following the extraction of features, serial based maximum value fusion is carried out, which is then enhanced using the moth flame optimization technique. Finally, machine learning classifiers such as support vector machines (SVM), neural networks, and others are used to classify the best optimal features. Below is a quick description of each sub-step.

### Dataset collection and normalization

**Figure 2:**
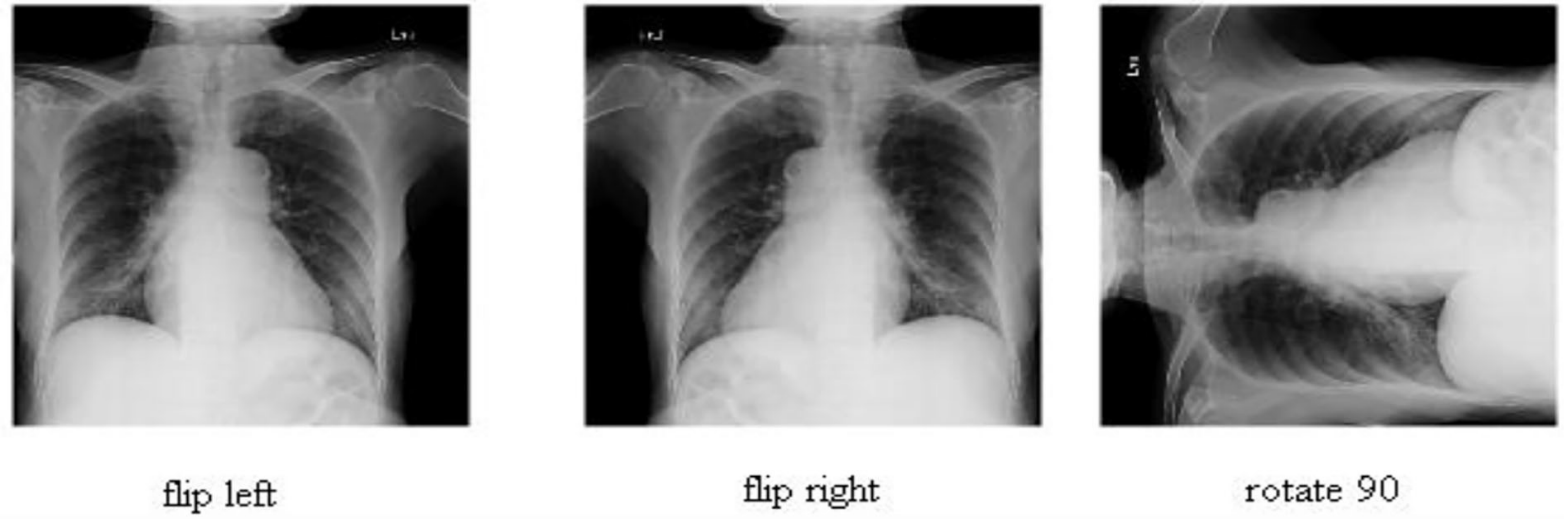
Sample images after the data augmentation step.

The experimental approach in this research uses three publicly available datasets: COVID-19 Radiography (https://www.kaggle.com/datasets/tawsifurrahman/covid19-radiography-database), COVID-GAN, and COVID-Net small chest x-ray (https://www.kaggle.com/yash612/covidnet-mini-and-gan-enerated-chest-xray), and Chest X-Ray (Pneumonia, COVID-19, Tuberculosis) (https://www.kaggle.com/datasets/jtiptj/chest-xray-pneumoniacovid19tuberculosis). There are four classes in the COVID-19 radiography dataset: COVID-19, lung opacity, normal, and viral pneumonia. COVID-19, normal, and pneumonia are the three classes in the COVID-GAN and COVID-Net small chest X-Ray dataset. COVID-19, normal pneumonia and tuberculosis are the four classes in the chest X-Ray dataset. The amount of images in each dataset is insufficient to train deep learning models, as shown in Table 1 for each dataset. Furthermore, these datasets are imbalanced, therefore we used data augmentation. Three simple approaches are used for data augmentation: rotate 90 degrees, flip left, and flip right. Figure 2 depicts the effects of each strategy graphically. The number of images is raised after the augmentation phase, as shown in Table 1.

**Table 1:** Brief description of selected datasets.

**Classes**	**Original images**	**Augmented images**	**Training/testing images**
**COVID-19 radiography database**			
COVID-19	3,616	5,864	2,932/2,932
Lung opacity	6,012	8,345	4,173/4,172
Normal	10,192	10,192	5,096/5,096
Viral pneumonia	1,345	4,976	2,488/2,488
**COVID-GAN and COVID-Net mini chest X-ray**			
Corona	461	3,119	1,560/1,559
Normal	1,575	3,048	1,524/1,524
Pneumonia	4.481	5,936	2,968/2,968
**Chest X-ray (pneumonia, COVID-19, tuberculosis)**			
COVID-19	566	3,396	1,698/1,698
Normal	1,575	3,150	1,575/1,575
Pneumonia	4,265	5,198	2,599/2,599
Tuberculosis	491	3,075	1,538/1,537

### Contrast enhancement

The enhancement of an input image is an important step to improve the quality of the original image. Based on this step, we obtained a brighter image (41). The primary motivation of this step in this study is to visualize the COVID-19 positive images than the healthy ones. The COVID-19 Radiography Database images have low contrast and bad quality; therefore, we designed a hybrid approach based on the fusion of different filtering outputs. Three different filters are opted such as top-hat and bottom-hat filtering, adjusting the pixel values, and sharp filter.

Consider that the COVID-19 Database has *n* images *D* ∈ ℝ^*n*^, where every image denoted by $k^{n}$(*x, y*) and (*x, y*) belong to ℝ. Every image has a size of N×M=512. Assume that s is a structuring element with the value of 11 and ◦ is an opening operator, • is a closing operator, then the top-hat and bottom-hat filtering is defined as follows:


(1) $$\begin{array}{l} k_{top}\left(x,y\right)=k^{n}\left(x,y\right)-\left(k^{n}\left(x,y\right)\;◦s\;\right) \end{array}$$

(2) $$\begin{array}{l} k_{bottom}\left(x,y\right)=\left(k^{n}\left(x,y\right)•\int \right)-k^{n}\left(x,y\right) \end{array}$$

(3) $$\begin{array}{l} f\left(x,y\right)=k^{n}\left(x,y\right)+k_{top}\left(x,y\right)-k_{bottom}\left(x,y\right) \end{array}$$

Where *f*(*x, y*) is the resultant top-bottom hat filtering image, k_*top*_(*x, y*) is the top-hat filtered image, and k_*bottom*_(*x, y*) is bottom hat filtered image, respectively. This image is further refined using adjust image pixel values filter. This filter raises the image's brightness by transferring the values of the input pixel intensity to particular values such that on average 1% of the information is absorbed in low and high input data intensities. The notation i is the intensity value of the image, and the *gamma (*γ*)* is a coefficient, which determines the form of the function between the coordinating variables (*a, c*) and (*b, d*).


(4) $$\begin{array}{l} A^{n}\left(x,y\right)={\left(\frac{i-a}{b-a}\right)}^{\gamma }\left(d-c\right)+c \end{array}$$

After that the resultant image $A^{n}\left(x,y\right)$ is sharpened using the unsharp masking method. This filter is applied to increase the contrast along the edges. The radius is 2 which specifies the length of the sharpness zone from around gray levels and the amount is 1 which leads to greater enhancement in the contrast of the enhanced pixels. The sharpen using an un-sharp mask denoted as:


(5) $$\begin{array}{l} g^{n}\left(x,y\right)=A^{n}\left(x,y\right)-k_{smooth}\left(x,y\right) \end{array}$$

(6) $$\begin{array}{l} S_{sharp}^{n}\left(x,y\right)=\;k^{n}\left(x,y\right)+a\times g^{n}\left(x,y\right) \end{array}$$

where k_*smooth*_(*x, y*) is a smoothed version of k^*n*^(*x, y*), $S_{sharp}^{n}\left(x,y\right)$ is a sharpen using the un-sharp mask filtered image and a is a scaling variable that provides the amount of sharpening. Hence, the resultant image is defined as follows:


(7) $$\begin{array}{l} R^{n}\left(x,y\right)=f\left(x,y\right)+A^{n}\left(x,y\right)+S_{sharp}^{n}\left(x,y\right) \end{array}$$

**Figure 3:**
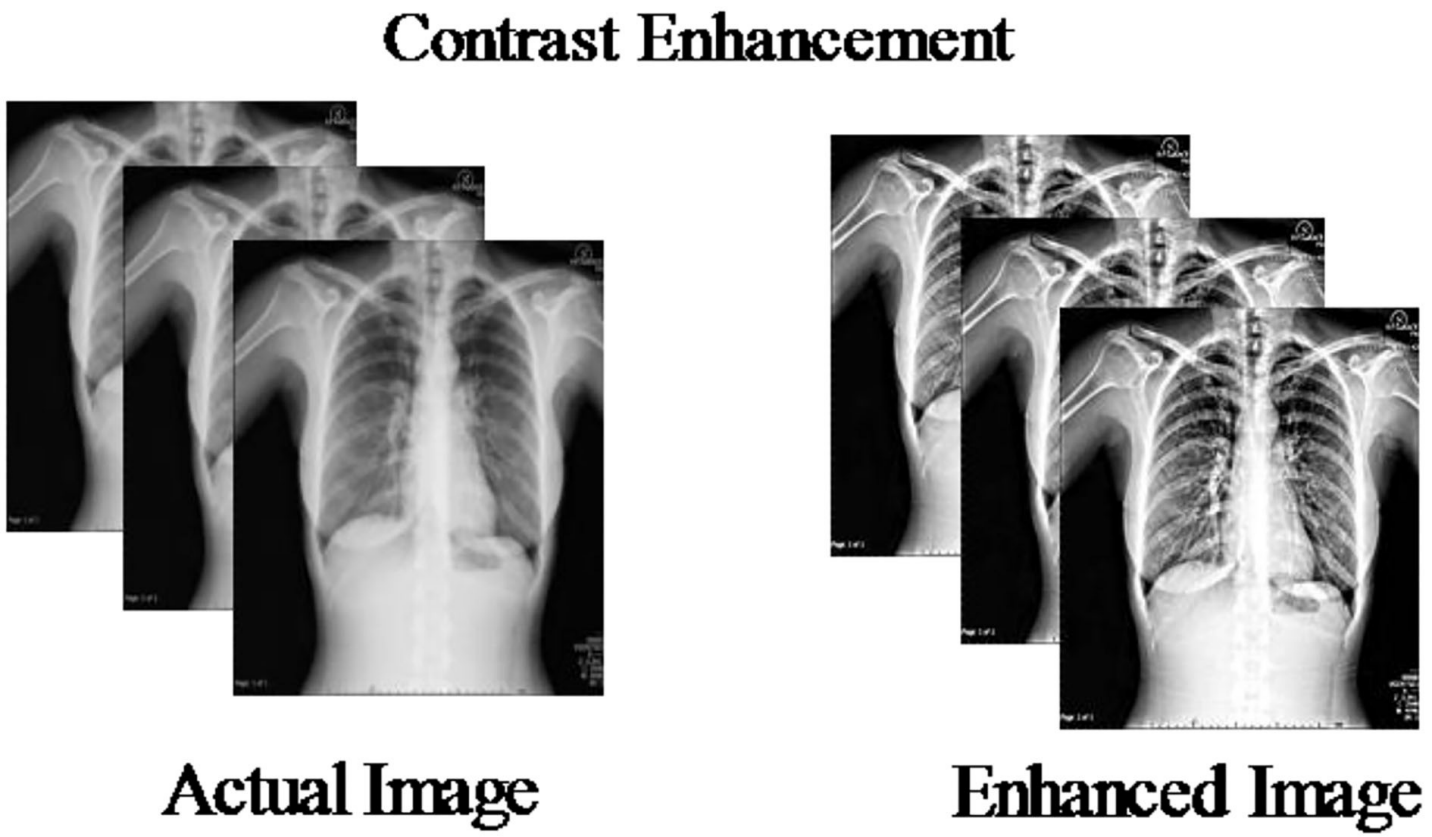
Sample resultant images of hybrid contrast enhancement technique.

Where *R*^*n*^(*x, y*) represents the resultant contrast enhanced image and is visually presented in Figure 3.

### EfficientNet deep features

EfficientNet is a deep neural network design and scaling method that uses a complicated parameter to evenly scale all depth, width, and resolution variables. Unlike current practice, which scales these variables arbitrarily, the EfficientNet scaling approach uses a pre-determined set of scalability variables to alter network breadth, depth, and resolution uniformly (42). This model was trained on 1,000 classes (ImageNet dataset) and accepts input images up to 224 × 224 × 3 pixels in size. As the fully-connected layer is replaced with a new fully-connected layer that includes the target classes, we fine-tune this model. The updated model was trained using a transfer learning strategy on the target datasets. Transfer learning (TL) is the process of reusing a previously learned model for a new task. In TL, a computer applies the information gained from previous work to improve prediction about a specific task. The primary goal of TL is to resolve the target domain efficiently. It is an excellent technique if the targeted domain dataset is much less than the origin domain dataset (43). Domain D={F,P(f)} Includes two parts: a higher dimensional space F and a marginal probability density P(f), where *F*= $\left\{f|f_{i}\in F,i=1,2\ldots .N\;\right\}$, and *N* is a dataset containing *M* items. Typically, distinct domains are established by the existence of distinct feature spaces or marginal probability distributions among them. when we give a specific domain D then it represented as: T={R,f(*)}. It also consists of two parts: a label space R and mapping function f(*) where $R=\left\{𝔯|{𝔯}_{i}\in R,i=1,2\ldots .N\right\}$ is a label space set which equivalent instances in D. The mapping procedure f(*), also represented as f(x)=P(f𝔯), is a non-linear and implicit procedure that may connect the input items to the projected choice, which is intended to be learned from the provided datasets.

**Figure 4:**
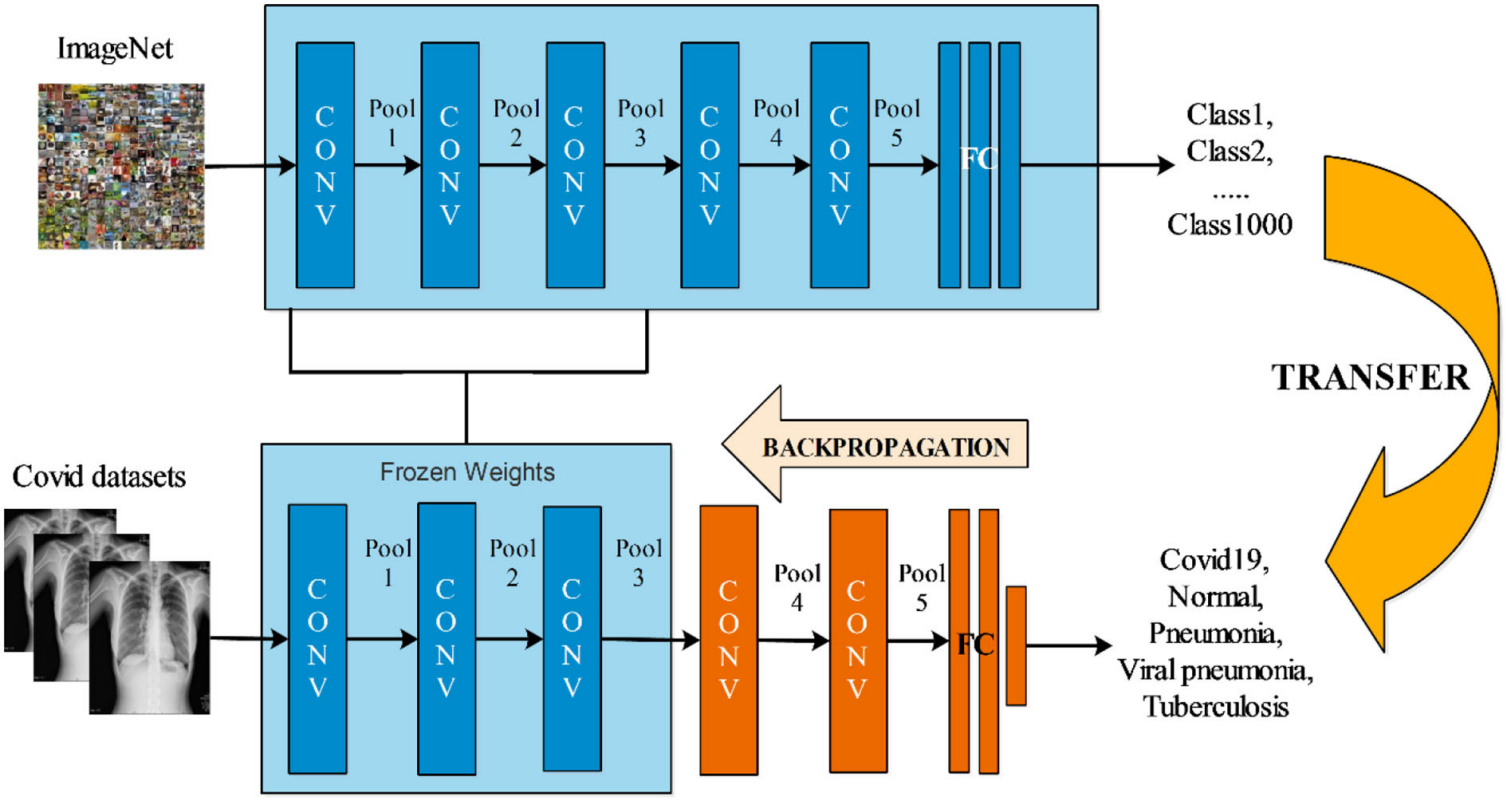
Visual process of transfer learning for the training of fine-tuned deep models.

Given an original domain $D^{o}=\left\{F^{o},P^{o}\left(f^{o}\right)\right\}$, with the original task $T^{o}=\left\{R^{o},f^{o}\left(*\right)\right\}$ and a target domain $D^{T}=\left\{F^{T},P^{T}\left(f^{T}\right)\right\}\;$ with target task $T^{T}=\left\{R^{T},f^{T}\left(*\right)\right\}$ intends to develop a more accurate mapping function $f^{T}\left(*\right)$ for the target task $T^{T}$ utilizing the transferrable knowledge acquired in the original domain $D^{o}$ and $T^{o}$. In contrast to traditional machine learning and deep learning, where the area and goal are similar across the original and target circumstances ($D^{o}=D^{T}\;\&\;T^{o}=\;T^{T}$). Transfer learning addresses issues that arise when the domain and task of the original and target situations are unrelated (i.e., $\left(D^{o}\neq D^{T}\text{ \& }T^{o}\neq \;T^{T}\right)$). Hence, the deep transfer learning can be expanded as: Given a transfer learning task $f^{o\to T}\left(*\right){:F}^{T}\to R^{T}$ based on $\left[D^{o},D^{T},T^{o},T^{T}\right]$. This process is visually illustrated in Figure 4. This figure illustrated how the original model weights and parameters are transferred to the updated model, which is subsequently trained using the COVID-19 datasets. After the training, deep features are extracted from the global average pooling layer of dimensional *N* × 1,280.

### LSTM-CNN features

LSTM is the category of recurrent neural network (RNNs), good at learning long-term relationships using residual connections (44). Feedforward neural networks have a “low memory” problem, which leads to poor performance on sequential and time-series tasks. To extract characteristics from time-series and sequence data, such models use cyclic linkages in their hidden layer. The well-known vanishing gradient difficulty hinders RNNs from learning long-term associations, despite this limitation. Input, output, and forget are the three basic gates in any LSTM network. As part of this framework, the LSTM learns long-term connections by “forgetting” and “preserving” information, allowing it to maintain a controlled flow of input (45). Further precisely, the input gate $I_{t}$ associate with the second gate ${𝔫}_{t}^{*}$ regulates the new knowledge that stored in the memory state 𝔫_*t*_ in time *t*. The forget gate $F_{t}$ regulates the previous knowledge which must be removed or retained in the memory block at time *t* − 1. Although, the output gate $Y_{t}$ determines which information may be used as the memory cell's output. Mathematically, it is defined as follows:


(8) $$\begin{array}{l} J_{t}=\sigma \left(M_{I}x_{t}+N_{I}h_{t-1}+b_{i}\right), \end{array}$$

(9) $$\begin{array}{l} F_{t}=\sigma \left(M_{g}x_{t}+N_{g}h_{t-1}+b_{g}\right), \end{array}$$

(10) $$\begin{array}{l} n_{t}^{*}=\tanh\left(M_{n}x_{t}+N_{n}h_{t-1}+b_{n}\right), \end{array}$$

(11) $$\begin{array}{l} n_{t}=g_{t}⊙n_{t-1}+J_{t}⊙n_{t}^{*}, \end{array}$$

(12) $$\begin{array}{l} y_{t}=\sigma \left(M_{y}x_{t}+N_{y}h_{t-1}+b_{y}\right), \end{array}$$

Where *x*_*t*_ represents the input, $M_{*}$ and $N_{*}$ are weights vectors and b_*_ are bias vectors, σ is the sigmoid procedure. ⊙ represents the element-wise multiplication. Finally, the hidden block *h*_*t*_ which includes the output of the memory block is evaluated by:


(13) $$\begin{array}{l} h_{t}=y_{t}⊙\tanh\left(n_{t}\right). \end{array}$$

**Figure 5:**
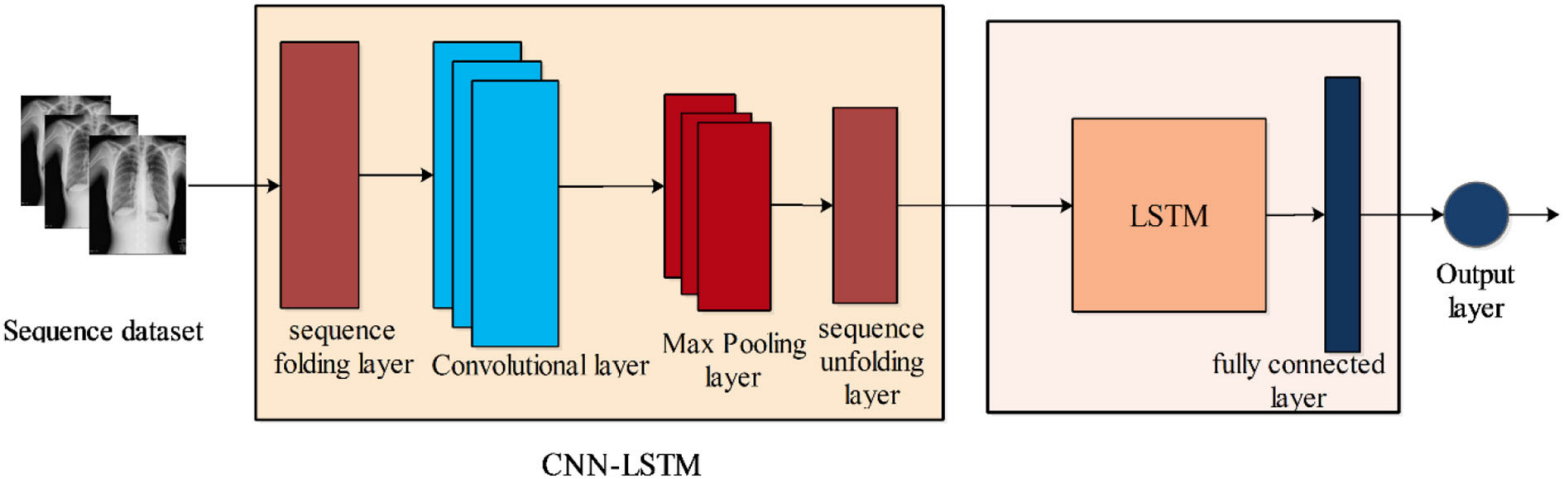
Proposed CNN-LSTM architecture.

In our study, we utilized the LSTM by using convolutional layers called CNN-LSTM. It consists of a convolutional layer of filter size is 5 and the number of filters is 20. Followed by a pooling layer, an LSTM layer included with the number of hidden units is 200. Furthermore, a fully connected layer, Softmax layer, and classification layers are added. The features are extracted from the LSTM layer and obtained a feature vector of dimension *N* × 200. Figure 5 illustrated the workflow of the proposed CNN-LSTM.

### Proposed features fusion

The process of combining information from two or more sources to improve an object's information is known as feature fusion. The serial-based features fusion technique is a simple but effective fusion method for combining data from multiple sources into a single matrix without losing any features. Simple serial based fusion is formulated as follows:

Consider that we have two feature vectors *f*_1_ and *f*_2_ having dimensions *N* × 1, 280 and *N* × 200, respectively. Then the serial based fusion vector dimension will be *N* × 1, 480 based on the following equation.


(14) $$\begin{array}{l} SFu\left(v\right)={\left[\begin{array}{c} f_{1}\\ f_{2} \end{array}\right]}_{N\times k_{1}+N\times k_{2}} \end{array}$$

This process combined all features in one matrix *SFu*(*v*) but after the analysis based on the results, it is observed that several of the combined features contain unrelated information; therefore, we tried to resolve this problem by employing a new equation called serial based maximum information.


(15) $$\begin{array}{l} mx=MAX\left(SFu\left(v\right)\right) \end{array}$$

(16) $$\begin{array}{l} V1=compare\left(f_{1},mx\right) \end{array}$$

(17) $$\begin{array}{l} V2=compare\left(f_{2},mx\right) \end{array}$$

(18) $$\begin{array}{l} S\tilde{F}u\left(v\right)={\left[\begin{array}{c} V1\\ V2 \end{array}\right]}_{N\times {\tilde{k}}_{1}+N\times {\tilde{k}}_{2}} \end{array}$$

Where $S\tilde{F}u\left(v\right)$ is the updated fused vector of dimension *N* × 980. This fused vector is further refined using the improved moth flame optimization approach.

### Features optimization

In the field of Computer Vision (CV), feature selection is the process of selecting the best subset of features from the original feature vector to improve the accuracy and reduce the computational time. The dimension of the solution space grows exponentially in proportion to the number of features in the data collection. As a result, exhaustive search strategies are unable to get the optimum solution in reality, and these feature selection techniques continue to struggle from a local optima standstill (46). The concept is that using a subset of features improves classifier performance and enables quicker classification, resulting in an equivalent or even better accuracy rate than when all features are used (47). In this study, we implemented an improved moth flame optimization (IMFO) for best feature selection. Consider, we have a fused feature vector of dimension *N* × 980 and after implementing the IMFO, the size of the resultant vector is *N* × 751.

In this algorithm, moths and flames are the main concepts and potential solutions are moths, that are based on the movements in space. Due to the population-based nature of the MFO algorithm, the set of *n* moths is employed as a search agent in the challenge space. Flames represent the best *n* locations of moths that have been discovered so far. As a result, each moth seeks for and updates a flame if a better solution is discovered. As a result, flames are *d* dimensional statistics as well. A particular moth updates the location based on the following formulation:


(19) $$\begin{array}{l} S\left(M_{m},F_{n}\right)=D_{m}\cdot e^{cr}\cdot \cos\left(2\pi r\right)+F_{n} \end{array}$$

Where *D*_*m*_ is the Euclidian Distance of the *m*^*th*^ moth for the *n*^*th*^ flame, *c* is the coefficient describing the shape of the logarithmic spiral, *M*_*m*_ represents the *m*^*th*^ moth, *F*_*n*_ represents the *n*^*th*^ flame and *r* is the random number between [−1, 1]. A moth's upcoming location is determined with a flame. As a result, a hyper-ellipse may be considered in all dimensions surrounding the flame, and the moth's new location will be contained inside this area. To stress exploitation, even more, t is a random integer in the range [*k*, 1], where *k* decreases linearly from 1 to 2 over the course of each iteration, referred to as the convergence rate. Along with increasing the likelihood of convergence to a global solution, each moth is required to update its location utilizing just one of the flames. Each iteration, and once the flames list has been updated, the flames are sorted according to their fitness values. The moths then adjust their locations concerning their assigned fires. To facilitate extensive exploitation of the most potential solution, the number of flames to be tracked is lowered in proportion to the number of iterations.


(20) $$\begin{array}{l} K_{flames}=round\left(K-l\cdot \frac{K-1}{Z}\right) \end{array}$$

Where *K* is the maximum number of flames, *l* is the current iteration number, and *Z* represents the maximum number of iterations. The selected K flames (features) are normalized and select the maximum values as follows:


(21) $$\begin{array}{l} NZ_{i}=\frac{K_{i}-\mu }{S} \end{array}$$

Where *K*_*i*_ denotes the selected flames, μ is the mean value, *S* denotes the standard deviation, and *NZ*_*i*_ is a normalized feature vector. After that, the max features are computed as follows:


(22) $$\begin{array}{l} Best=\underset{1<i\leq \tilde{nr}}{\max}\left(NZ_{i}\right) \end{array}$$

Where, $\tilde{nr}$ represent the maximum iterations (100 times). Finally, we set a comparison among *K*_*i*_ and best selected features by Equation (23) and the best vector. The quadratic SVM is opted as a fitness function and the performance of the fitness function is measured through the mean squared error rate (MSER). The final selection is defined as follows:


(23) $$FS_{i}=\{\begin{array}{c} \tilde{FS}\left(i\right)\;for\;Best\geq T\\ Discard,\;Elsewhere \end{array},\;where\;T=0.4$$

## Results and analysis

The detailed experimental results of the proposed framework have been presented in this section- tabular form and visual graphs. Three datasets have been utilized for the experimental process and detail of each dataset has been given under Section Dataset collection and normalization. The results of each dataset are presented separately under the below sections. Each dataset is divided into 50:50 and set cross-validation value to 10. Several classifiers are utilized for the classification comparison and each classifier's performance is opted using several measures such as sensitivity rate, precision rate, F1-Score, accuracy, and time. The entire experimental process is tested on MATLAB2021b using Personal Desktop Computer with 16 GB of RAM and an 8 GB graphics card.

### COVID-19 radiography database results

#### Proposed fusion

**Figure 6:**
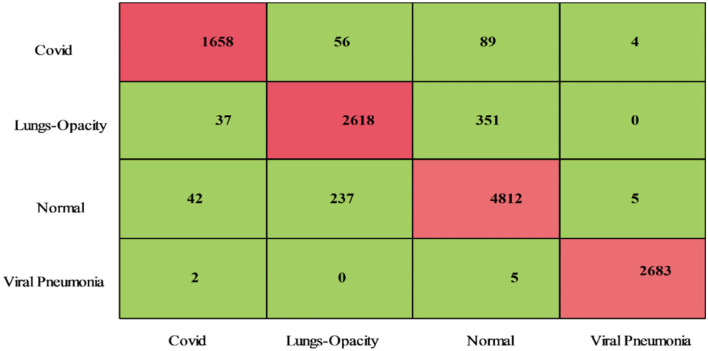
Confusion matrix of QSVM using proposed fusion method on COVID-19 Radiography Database.

**Figure 7:**
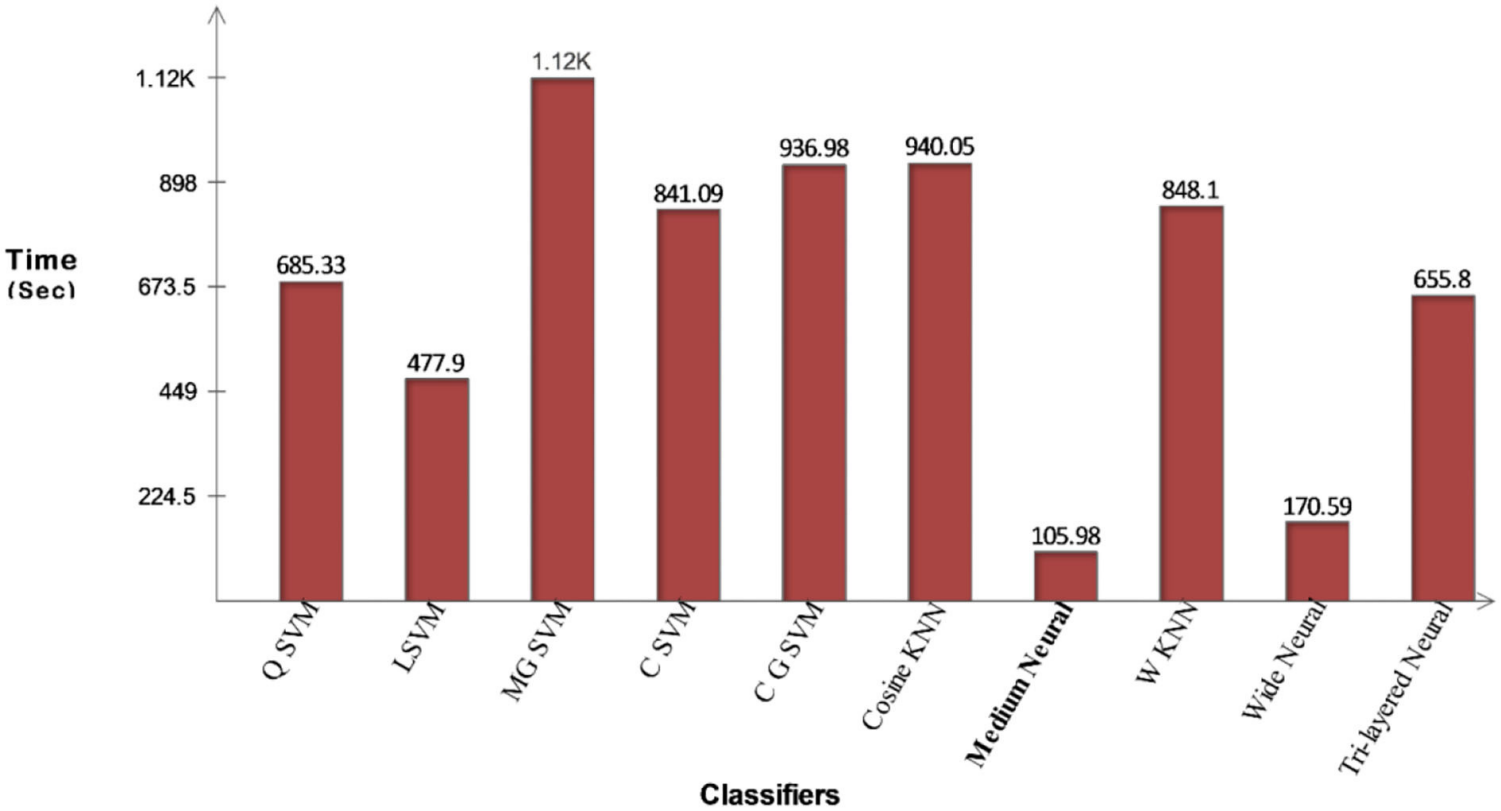
Illustration of computational time for COVID-19 Radiography Database after proposed fusion method.

The proposed fusion method classification results for the COVID-19 Radiography dataset have been presented in Table 2. This table presents that the maximum attained accuracy is 93.4% for the QSVM classifier. For this classifier, the noted sensitivity rate is 93.25%, the precision rate is 94.12%, F1-Score is 93.68%, FPR is 0.025, and AUC is 0.98. These values are also computed for the rest of the classifiers and it is observed based on the numerical values, the performance of QSVM is better than the rest of the classifiers. These computed measures of the QSVM classifier can be further verified using a confusion matrix, illustrated in Figure 6. The computational time of each classifier is noted for this experiment and the minimum time is 105.98 (s) for the Medium Neural classifier, whereas, the maximum execution time is 1,122.5(s). Moreover, a clear picture of the change in time of different classifiers is shown in Figure 7. From this figure, it is observed that the Medium Neural classifier needs less time for execution than the rest of the classifiers.

**Table 2:** Proposed features fusion method results on COVID-19 Radiography Database.

**Classifiers**	**Sensitivity**	**Precision**	**FPR**	**AUC**	**F1-score**	**Accuracy**	**Time (s)**
Q SVM	**93.25**	**94.12**	**0.025**	**0.98**	**93.68**	**93.4**	**685.33**
L SVM	92.87	93.81	0.025	0.98	93.33	93.2	477.9
M G SVM	92.72	93.92	0.025	0.987	93.31	93.1	1,122.5
C SVM	93.12	94.00	0.025	0.98	93.55	93.2	841.09
C G SVM	91.82	93.07	0.027	0.98	92.44	92.4	936.98
Cosine KNN	91.52	91.61	0.032	0.98	91.55	91.3	940.05
Medium neural	91.87	92.00	0.03	0.97	91.93	91.5	105.98
W KNN	90.9	90.63	0.032	0.98	90.76	90.6	848.1
Wide neural	92.25	92.57	0.027	0.98	92.40	92.0	170.59
Tri-layered neural	91.25	91.02	0.0325	0.96	91.13	90.8	655.8

Bold indicates the best accuracy.

#### Proposed IMFO

**Figure 8:**
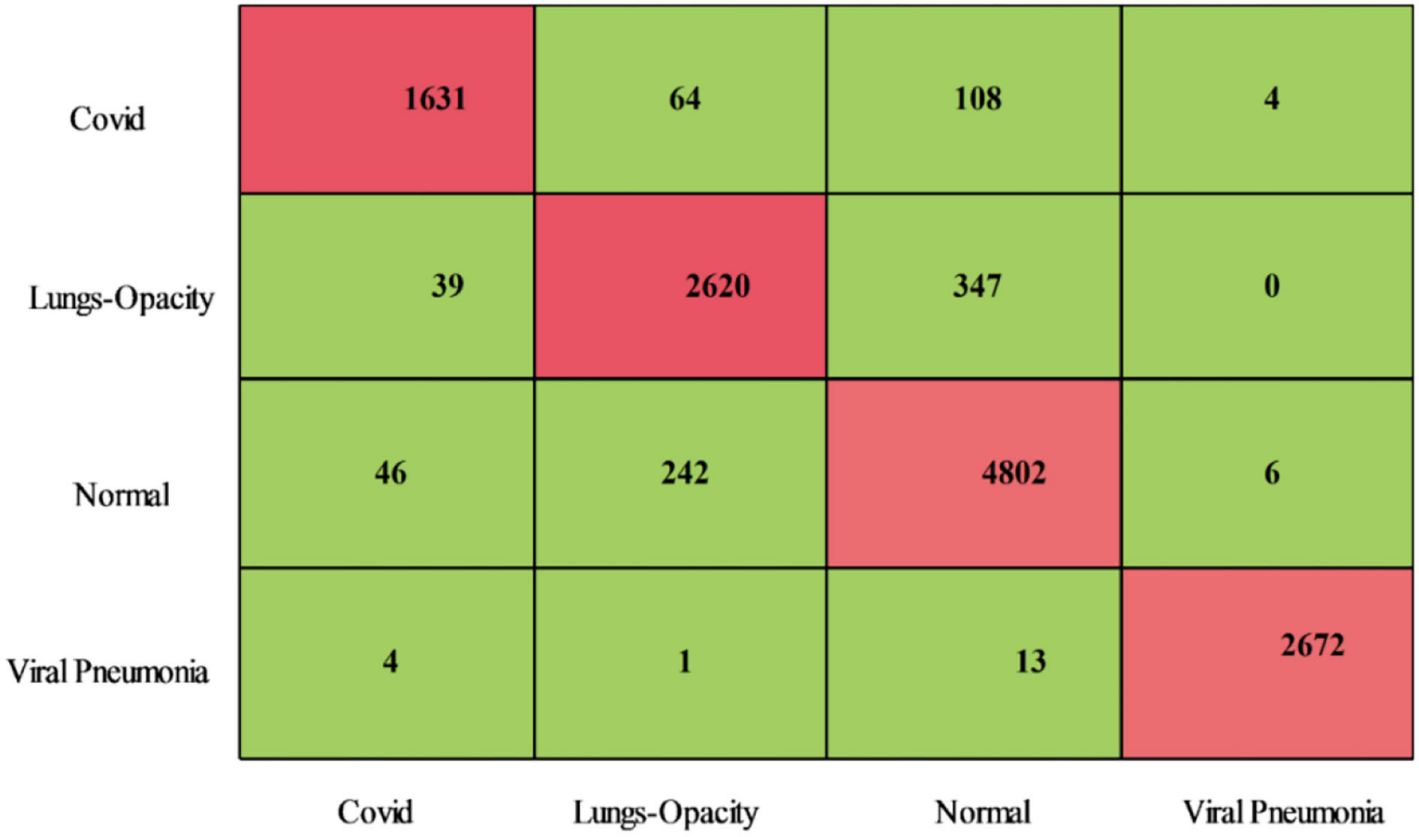
Confusion matrix of CSVM using proposed optimization method on COVID-19 Radiography Database.

**Figure 9:**
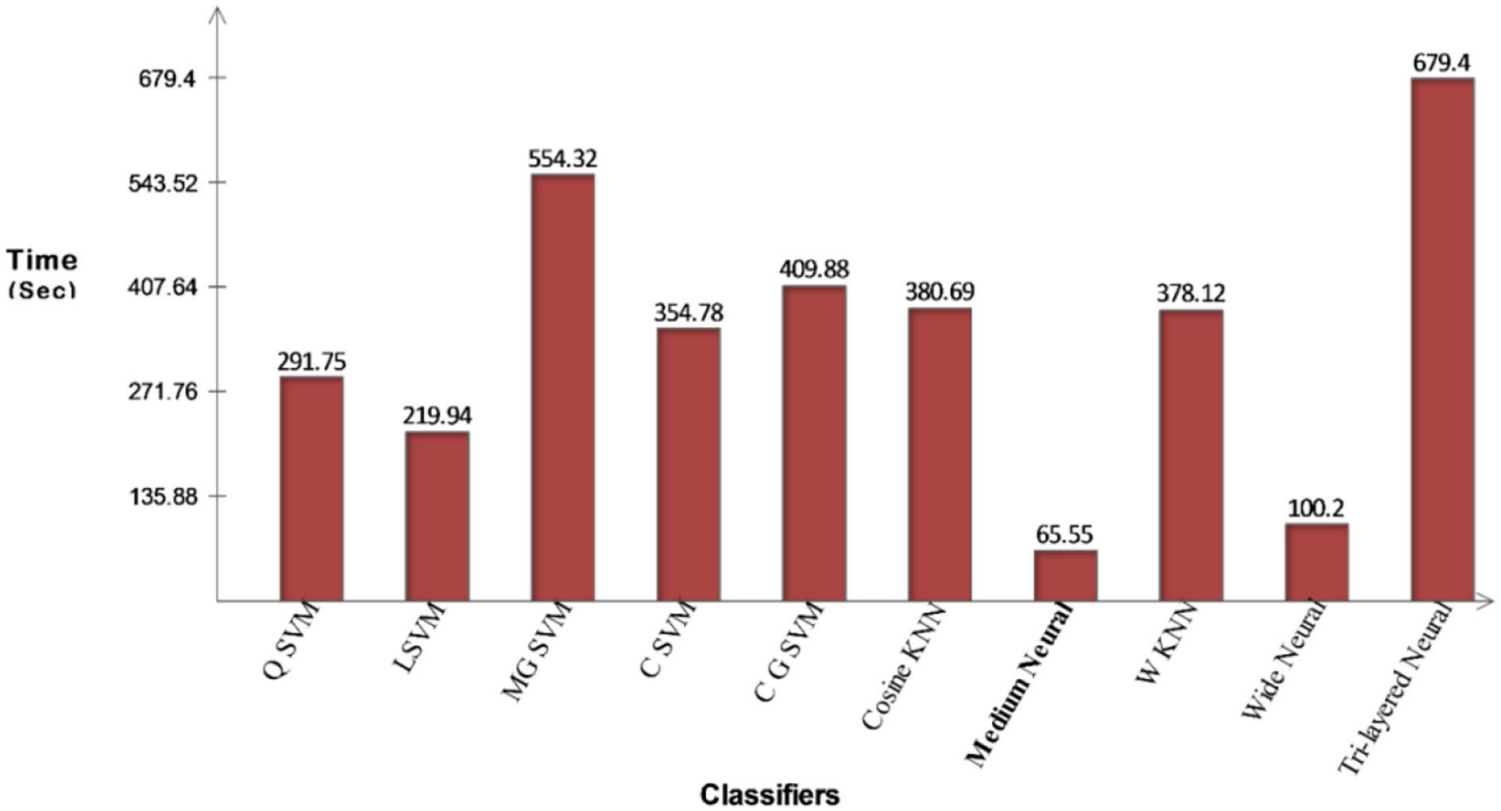
Illustration of computational time for COVID-19 Radiography Database after proposed optimization method.

The proposed optimization results for the COVID-19 Radiography dataset have been presented in Table 3. This table presents the best accuracy of 93.0% for the CSVM classifier. For this classifier, highlighted sensitivity rate is 92.82%, the precision rate is 93.00%, F1-Score is 92.90%, FPR is 0.025, and AUC is 0.98. These values are likewise calculated for the other classifiers, and it is noted that CSVM performs better than the remaining classifiers based on the statistical figures. This CSVM classifier's generated scores could be further confirmed using a confusion matrix, illustrated in Figure 8. For this hypothesis, the computing time of each classifier is recorded; the quickest time is 65.55 (s) for the Medium Neural classifier, while the highest execution time is 554.32 (s). Additionally, Figure 9 illustrates the relationship between the change in time of several classifiers. As shown in this figure, the Medium Neural classifier requires less time to execute than the other classifiers.

**Table 3:** Proposed features optimization results on COVID-19 Radiography Database.

**Classifiers**	**Sensitivity**	**Precision**	**FPR**	**AUC**	**F1-score**	**Accuracy**	**Time (s)**
Q SVM	92.85	93.05	0.025	0.98	92.94	92.9	291.75
L SVM	92.65	93.06	0.025	0.98	92.85	92.9	219.94
M G SVM	92.72	92.03	0.025	0.98	92.37	92.3	554.32
C SVM	**92.82**	**93.00**	**0.025**	**0.98**	**92.90**	**93.0**	**354.78**
C G SVM	91.67	93.07	0.027	0.98	92.36	92.3	409.88
Cosine KNN	91.82	91.82	0.032	0.98	91.82	91.6	380.69
Medium neural	91.07	92.12	0.03	0.97	91.59	90.7	65.55
W KNN	90.85	91.17	0.03	0.98	91.00	90.8	378.12
Wide neural	91.07	91.92	0.03	0.97	91.49	91.5	100.2
Tri-layered neural	90.62	90.6	0.032	0.95	90.60	90.3	679.4

Bold indicates the best accuracy.

### COVID-GAN and COVID-Net mini chest X-ray dataset

#### Fusion method

**Figure 10:**
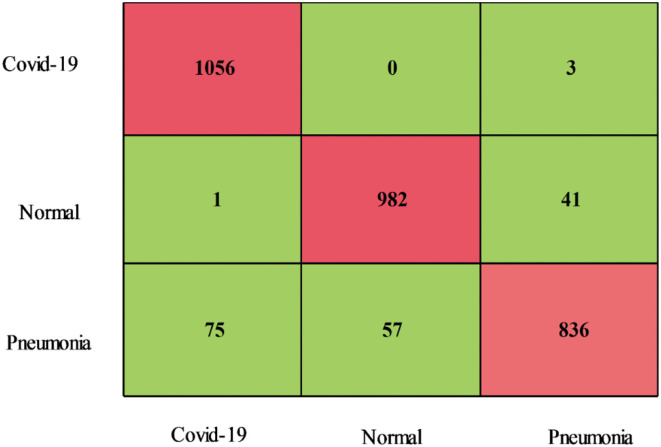
Confusion matrix of COVID-GAN and COVID-Net mini chest X-ray dataset after fusion.

**Figure 11:**
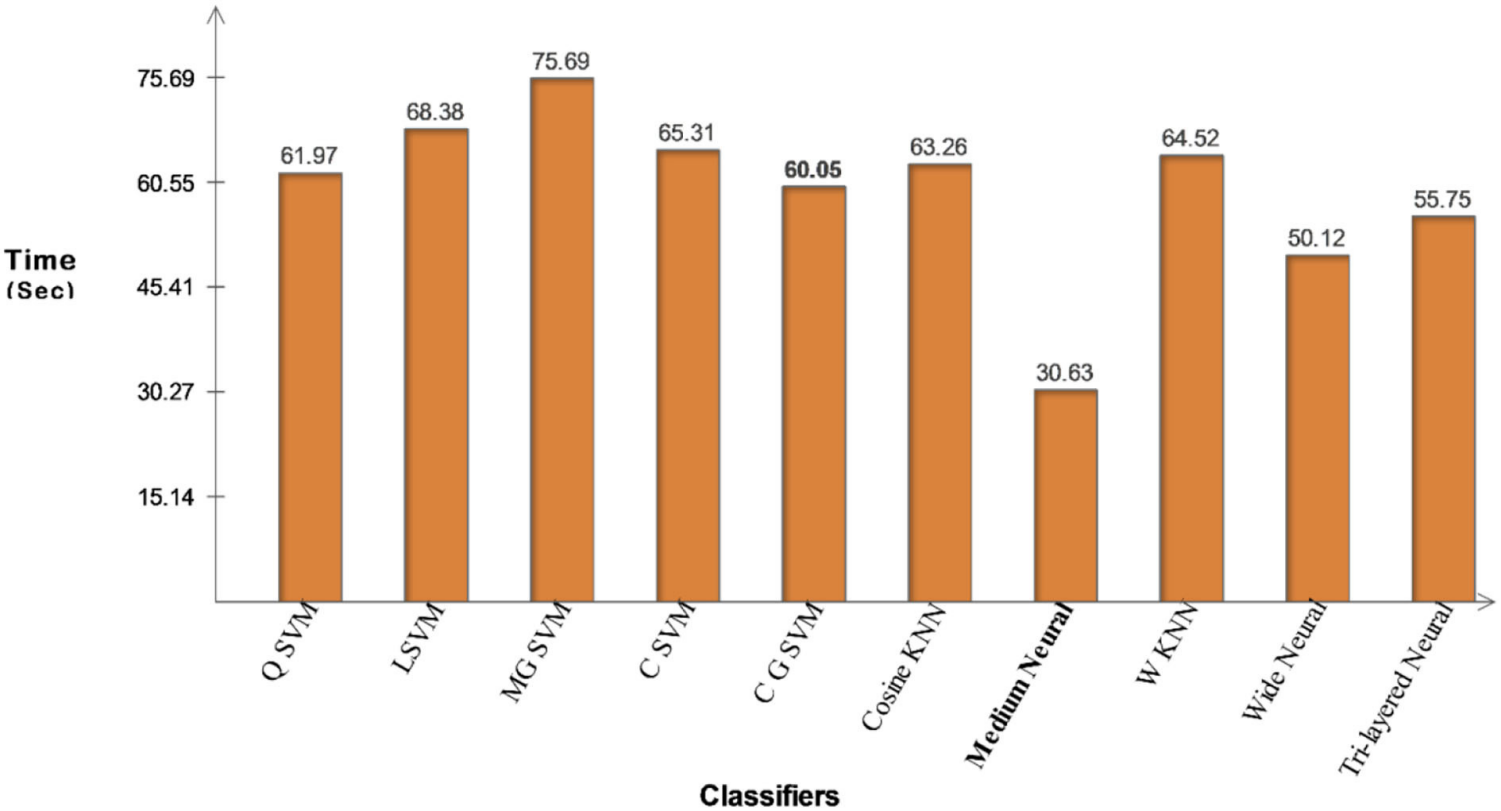
Illustration of computational time for COVID-GAN and COVID-Net mini chest X-ray dataset after proposed fusion method.

The classification results of the proposed fusion method using the Chest X-Ray dataset are presented in Table 4. This table illustrates that the CSVM classifier best accuracy of 94.2 %, sensitivity is 94.00 %, precision rate is 94.26 %, F1-Score is 94.12 %, FPR is 0.03, and AUC is 0.99 for this classifier. CSVM outperforms the rest of the classifiers in terms of statistical numbers, as are the values generated for the other classifiers. A confusion matrix is also illustrated in Figure 10 for the confirmation of CSVM performance. Classifier computation times have been noted for each classifier and the best noted time is 30.626 (s) for the Medium Neural classifier, whereas the highest execution time is 75.685 (s). Figure 11 depicts the execution time of all selected classifiers and shows that the Medium Neural Network classifier takes less time.

**Table 4:** Proposed features fusion method results on COVID-GAN and COVID-net mini chest X-ray dataset.

**Classifiers**	**Sensitivity**	**Precision**	**FPR**	**AUC**	**F1-score**	**Accuracy**	**Time (s)**
Q SVM	94.46	93.76	0.03	0.98	94.10	93.7	61.969
L SVM	93.00	93.33	0.03	0.98	93.16	93.3	68.377
M G SVM	93.10	93.43	0.03	0.98	93.26	93.4	75.685
C SVM	**94.00**	**94.26**	**0.03**	**0.99**	**94.12**	**94.2**	**65.307**
C G SVM	91.96	92.37	0.03	0.97	92.16	92.3	60.051
Cosine KNN	89.10	90.46	0.05	0.97	89.77	89.5	63.264
Medium neural	93.70	93.90	0.03	0.98	93.79	93.9	30.626
W KNN	93.72	93.90	0.03	0.98	93.80	92.1	64.517
Wide neural	93.63	93.9	0.03	0.98	93.76	93.8	50.115
Tri-layered neural	92.46	92.63	0.03	0.96	92.54	92.7	55.752

Bold indicates the best accuracy.

#### Proposed IMFO

**Figure 12:**
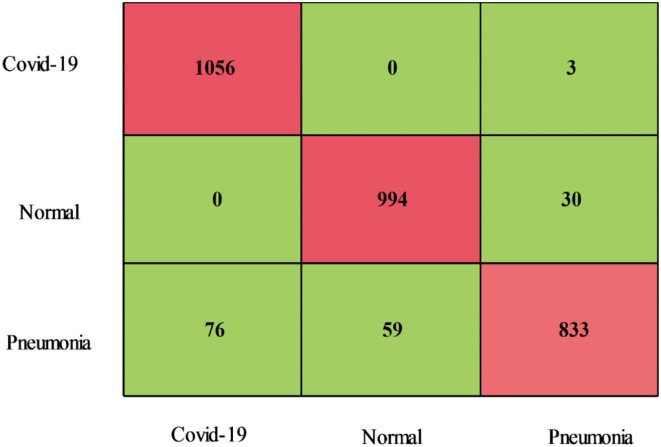
Confusion matrix of COVID-GAN and COVID-Net mini chest X-ray dataset using proposed optimization method.

**Figure 13:**
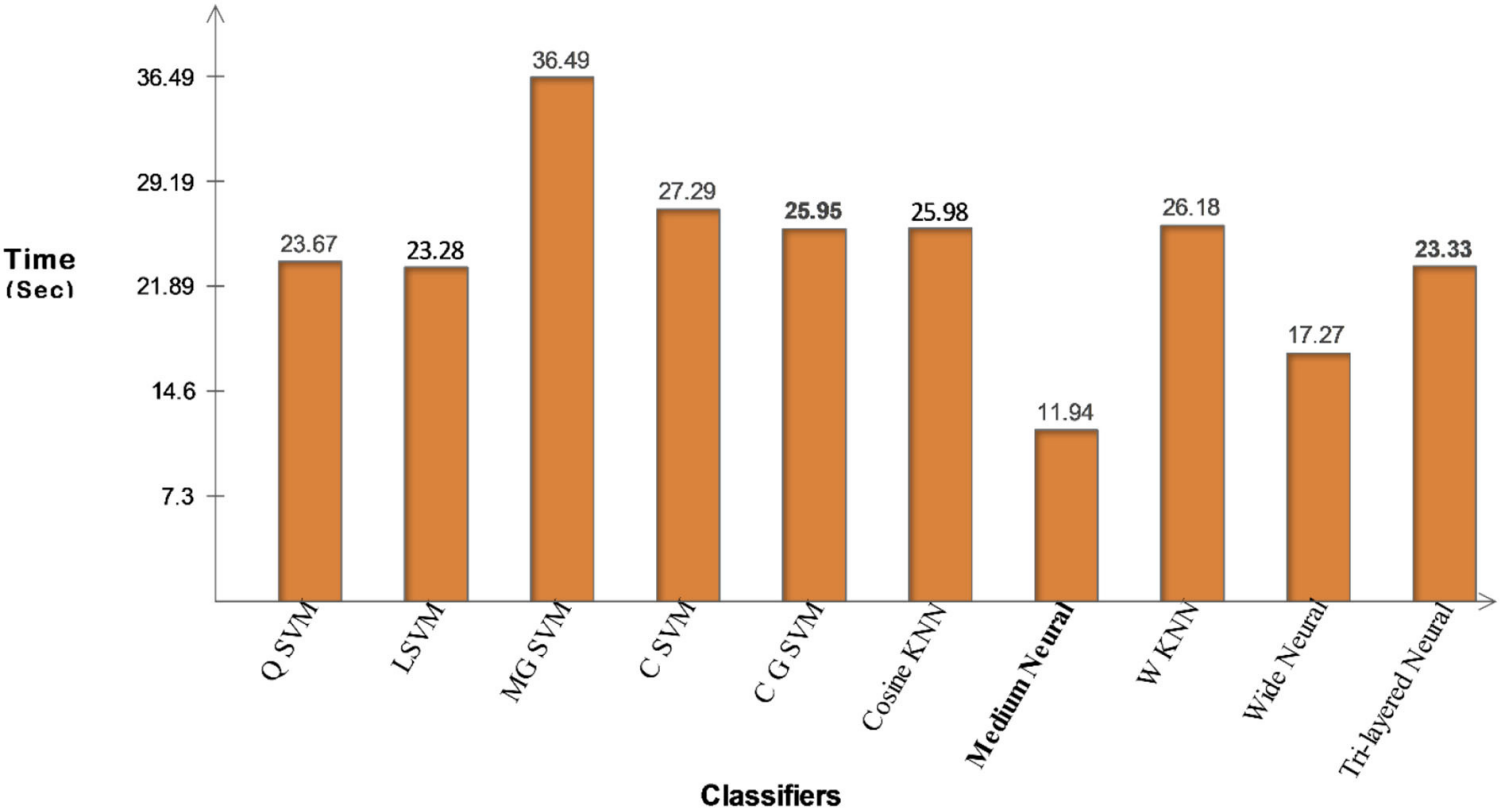
Illustration of computational time for COVID-GAN and COVID-Net mini chest X-ray dataset after proposed optimization method.

The proposed features optimization results on COVID-GAN and COVID-Net mini Chest X-ray dataset have been presented in Table 5. The CSVM classifier attained an accuracy of 94.5%, whereas the sensitivity of 94.30, precision rate of 94.63, an F1-Score of 94.46%, an FPR of 0.03, and an AUC is 0.99, respectively. Figure 12 illustrates the confusion matrix of CSVM for the confirmation of computed values. We also noted the classifiers' computational time during the testing process and the Medium Neural classifier has the shortest time duration of 11.943 (s), whereas the highest execution time is 36.492 (s). The computational time of each classifier is also plotted in Figure 13.

**Table 5:** Proposed features optimization method results on COVID-GAN and COVID-Net mini chest X-ray dataset.

**Classifiers**	**Sensitivity**	**Precision**	**FPR**	**AUC**	**F1-score**	**Accuracy**	**Time (s)**
Q SVM	93.80	94.13	0.03	0.98	93.96	94.0	23.666
L SVM	92.96	93.30	0.03	0.98	93.12	93.2	23.28
M G SVM	93.26	93.60	0.03	0.98	93.42	93.5	36.492
C SVM	**94.30**	**94.63**	**0.03**	**0.99**	**94.46**	**94.5**	**27.285**
C G SVM	91.96	92.2	0.04	0.97	92.07	92.1	25.952
Cosine KNN	91.23	91.70	0.04	0.97	91.46	91.6	25.98
Medium neural	93.33	93.53	0.03	0.98	93.42	93.5	11.943
W KNN	91.90	92.60	0.04	0.97	92.24	92.2	26.175
Wide neural	93.66	93.93	0.03	0.98	93.79	93.9	17.266
Tri-layered neural	92.43	92.60	0.03	0.96	92.51	92.7	23.331

Bold indicates the best accuracy.

### Chest X-ray dataset (pneumonia, COVID-19, tuberculosis)

#### Fusion results

**Figure 14:**
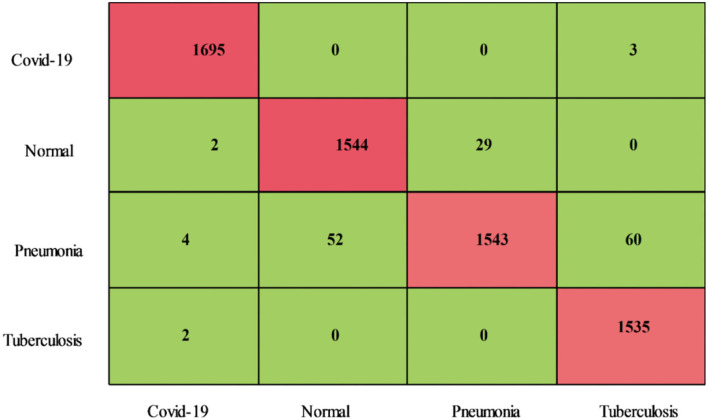
Confusion matrix of chest X-ray dataset for proposed fusion method.

**Figure 15:**
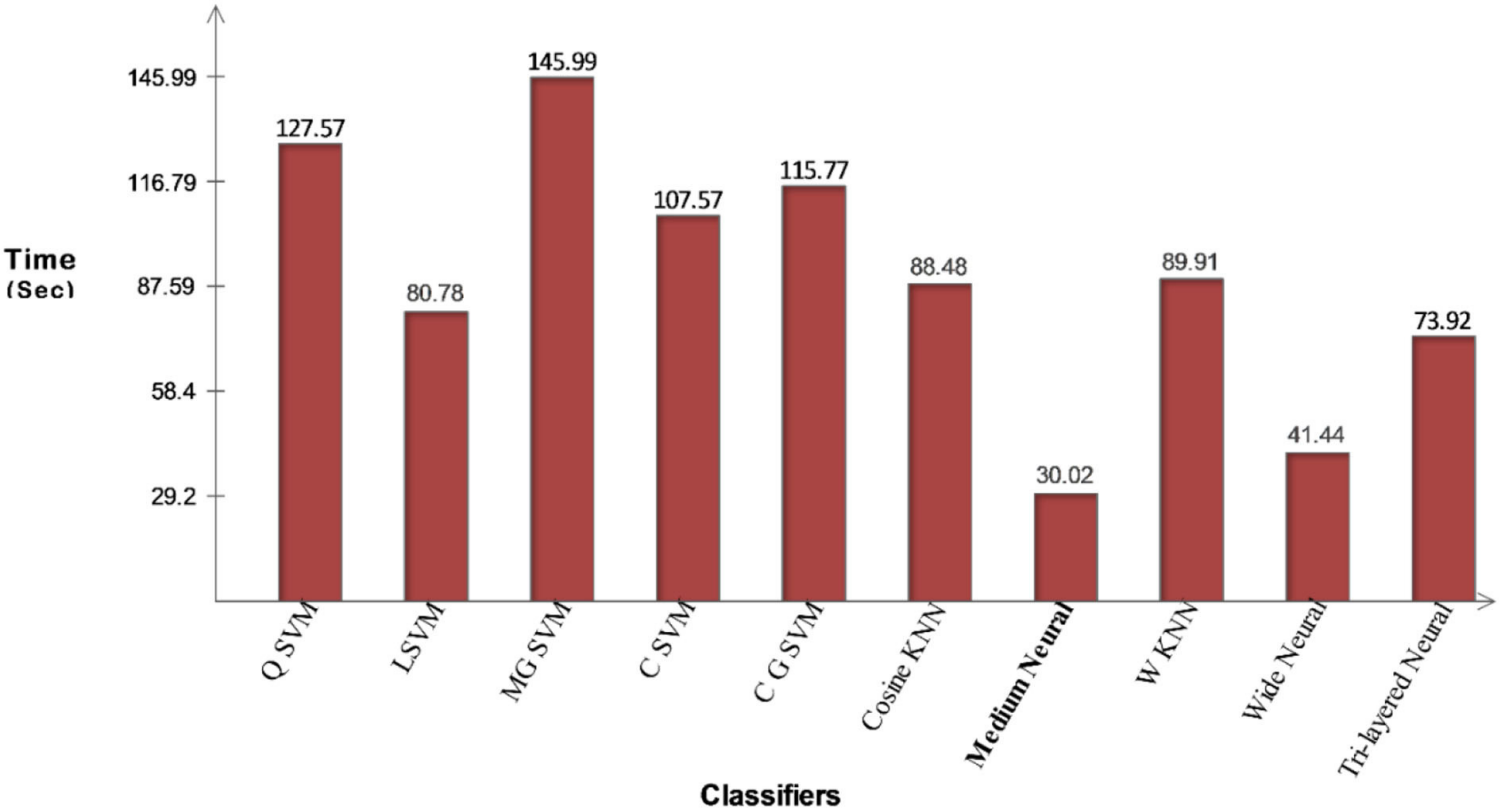
Illustration of computational time for chest X-ray dataset using the proposed fusion method.

Classification results for the Chest X-Ray (Pneumonia, COVID-19, Tuberculosis) dataset have been shown in Table 6. This table shows that the CSVM classifier attained the best accuracy of 98.3%. Among the other calculated measures, the sensitivity rate is 98.32, the precision rate is 98.32%, the F1-Score is 98.32%, the FPR is 0.05, and the AUC is 1. These values are also calculated for the other classifiers, and based on the numerical values, it is noted that the CSVM outperforms other classifiers. Figure 14 illustrated the confusion matrix of CSVM that was utilized for the confirmation of calculated values. For each classifier, the execution time is also noted, as plotted in Figure 15. In this figure, it is shown that the minimum noted is 30.022 s for the Medium Neural classifier, whereas the maximum noted time is 145.99 (s) for the MGSVM.

**Table 6:** Proposed features fusion method results on chest X-ray dataset.

**Classifiers**	**Sensitivity**	**Precision**	**FPR**	**AUC**	**F1-score**	**Accuracy**	**Time (s)**
Q SVM	98.32	98.32	0.05	1.00	98.32	98.3	127.57
L SVM	97.82	97.85	0.05	1.00	97.83	97.8	80.783
M G SVM	97.92	97.95	0.05	1.00	97.93	97.9	145.99
C SVM	**98.32**	**98.32**	**0.05**	**1.00**	98.32	**98.3**	**107.57**
C G SVM	96.72	96.70	0.012	1.00	96.70	96.6	115.77
Cosine KNN	90.87	91.65	0.027	0.99	91.26	90.7	88.484
Medium neural	97.92	97.90	0.007	1.00	97.91	97.9	30.022
W KNN	95.92	96.15	0.015	0.99	96.03	95.9	89.907
Wide neural	98.02	98.05	0.005	1.00	98.03	98.0	41.439
Tri-layered neural	97.80	97.77	0.007	0.99	97.78	97.8	73.92

Bold indicates the best accuracy.

#### IMFO method

**Figure 16:**
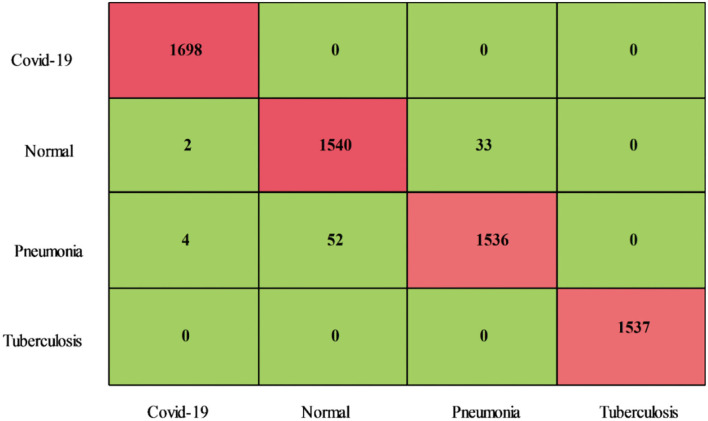
Confusion matrix of chest X-ray dataset for proposed optimization method.

**Figure 17:**
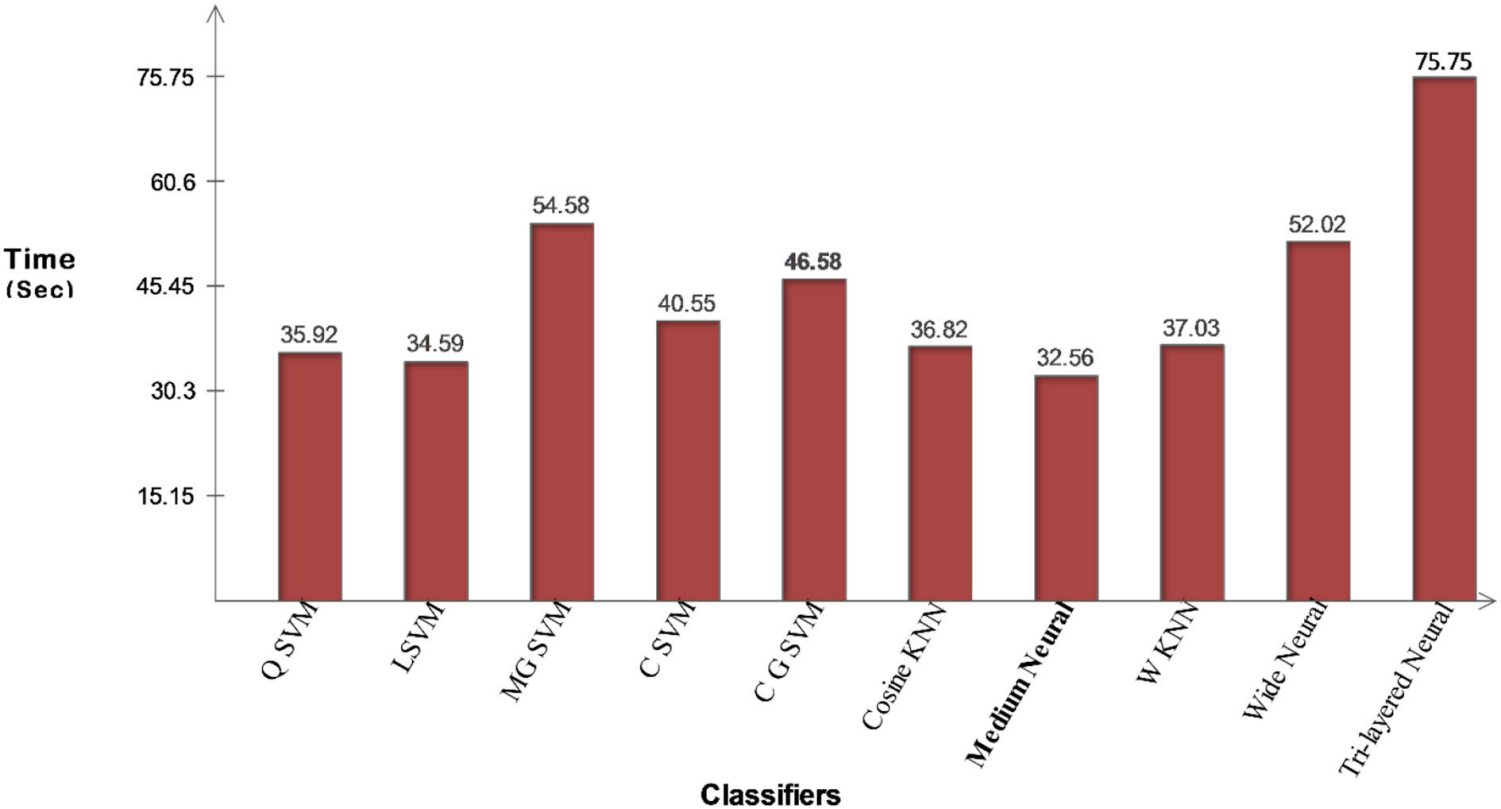
Illustration of computational time for chest X-ray dataset using proposed optimization method.

The proposed optimization method based classification results are given in Table 7. This table demonstrates that the CSVM classifier has attained the best accuracy of 98.5%. The values of other measures such as sensitivity rate is 98.50, the precision value is 98.22, F1-Score is 98.35%, FPR is 0.005, and AUC is 1, respectively. These statistics are also generated with the other learners, and based on the statistical results, it is reported that CSVM beats other listed classifiers. The CSVM performance can be further confirmed using a confusion matrix, illustrated in Figure 16. The execution time of each classifier is also noted and the minimum time is 32.537 (s) for the Medium Neural classifier. The maximum reported time is 75.75 (s) for the Tri-layered neural network. The time of all classifiers is also plotted in Figure 17. Overall, it is observed that the proposed optimization method performed well for all selected datasets.

**Table 7:** Proposed features optimization method results on chest X-ray dataset.

**Classifiers**	**Sensitivity**	**Precision**	**FPR**	**AUC**	**F1-score**	**Accuracy**	**Time (s)**
Q SVM	98.00	98.25	0.005	1.00	98.12	98.3	35.919
L SVM	97.75	97.75	0.005	1.00	97.75	97.9	34.585
M G SVM	97.50	97.75	0.005	1.00	97.62	97.9	54.583
C SVM	**98.50**	**98.22**	**0.005**	**1.00**	**98.35**	**98.5**	**40.551**
C G SVM	96.50	96.50	0.012	1.00	96.50	96.6	46.575
Cosine KNN	96.00	96.25	0.012	0.99	96.12	96.1	36.823
Medium neural	97.92	97.90	0.005	1.00	97.90	98.1	32.537
W KNN	96.00	96.25	0.015	0.99	96.12	95.9	37.033
Wide neural	98.01	98.12	0.005	1.00	98.06	98.1	52.025
Tri-layered neural	97.95	98.00	0.005	0.99	97.97	98.0	75.75

Bold indicates the best accuracy.

In the end, a detailed comparison is conducted with some recent techniques, presented in Table 8. In this table, several recently published techniques have been given and all of them used the deep learning framework. Recently, the maximum noted accuracy is 98.1% by (50). However, the proposed framework achieved an accuracy of Table 8, we compare the proposed technique with the different deep learning techniques. The COVID-19 diagnosis using deep learning methods on chest X-ray images was proposed and this technique achieved high accuracy of 94.7%. Classification of chest x-ray images using deep learning approaches and achieved 96.1% accuracy. The detection of COVID-19 by using deep learning through chest CT images for the joint edge-cloud computing framework method achieved 96.4%. A method based on deep learning for automatically diagnosing COVID-19 images using chest X-ray images acquired 96.6% accuracy. By using LSTM with an attention mechanism for COVID-19 detection and nodules segmentation on chest CT scans, the technique gained high accuracy of 98.1%. Our proposed method achieves 98.5%.

**Table 8:** Comparison of the proposed framework with recent techniques.

**Sr. no**	**References**	**Year**	**Method**	**Accuracy (%)**
1	(24)	2020	Deep learning based technique	94.7
2	(32)	2022	Deep CNN method	96.1
3	(48)	2021	Deep learning and collaborative edge-cloud computing	96.4
4	(49)	2022	Deep learning method	96.6
5	(50)	2022	LSTM with attention mechanisms	98.1
Proposed			CNN-LSTM and fusion-optimization	93.0
				94.5
				**98.5**

Bold indicates the best accuracy.

## Conclusion

We proposed an automated deep learning and improved optimization algorithm-based framework for COVID-19 classification using Chest X-ray images in this paper. In the proposed framework, contrast enhancement is done first to improve the quality of the infected region, and then data augmentation is used to increase the training samples. Following that, a CNN-LSTM architecture is created and trained with deep transfer learning. In addition, an EfficientNet deep model is fine-tuned, and feature extraction for both developed models is performed. Later, instead of using the original serial-based approach, the proposed fusion approach is used to better combine the information. The analysis of the fused feature vector reveals several redundant features; thus, a new features optimization technique is proposed. The proposed optimization method improves accuracy while also shortening classification time. The controlled vector size during the fusion process is the work's limitation. Furthermore, the optimization technique appears to have reduced some important features, which may have resulted in a reduction in final accuracy.

## Data availability statement

Publicly available datasets were analyzed in this study. This data can be found here: COVID-19 Radiography (https://www.kaggle.com/datasets/tawsifurrahman/covid19-radiography-database), COVID-GAN and COVID-Net small chest x-ray (https://www.kaggle.com/yash612/covidnet-mini-and-gan-enerated-chest-xray), and Chest X-Ray (Pneumonia, COVID-19, Tuberculosis) (https://www.kaggle.com/datasets/jtiptj/chest-xray-pneumoniacovid19tuberculosis).

## Author contributions

All authors listed have made a substantial, direct, and intellectual contribution to the work and approved it for publication.

## Funding

The authors extend their appreciation to the Deanship of Scientific Research at King Khalid University for supporting this study through the Large Groups Project under grant number (RGP.2/16/43).

## Conflict of interest

The authors declare that the research was conducted in the absence of any commercial or financial relationships that could be construed as a potential conflict of interest.

## Publisher's note

All claims expressed in this article are solely those of the authors and do not necessarily represent those of their affiliated organizations, or those of the publisher, the editors and the reviewers. Any product that may be evaluated in this article, or claim that may be made by its manufacturer, is not guaranteed or endorsed by the publisher.
